# PD-1 Ligand Expression in Epithelial Thyroid Cancers: Potential Clinical Implications

**DOI:** 10.3390/ijms20061405

**Published:** 2019-03-20

**Authors:** Salvatore Ulisse, Chiara Tuccilli, Salvatore Sorrenti, Alessandro Antonelli, Poupak Fallahi, Eleonora D’Armiento, Antonio Catania, Francesco Tartaglia, Maria Ida Amabile, Laura Giacomelli, Alessio Metere, Nicola Cornacchini, Daniele Pironi, Giovanni Carbotta, Massimo Vergine, Massimo Monti, Enke Baldini

**Affiliations:** 1 Department of Surgical Sciences, “Sapienza” University of Rome, 00161 Rome, Italy; salvatore.ulisse@uniroma1.it (S.U.); chiara.tuccilli@gmail.com (C.T.); salvatore.sorrenti@uniroma1.it (S.S.); antonio.catania@uniroma1.it (A.C.); francesco.tartaglia@uniroma1.it (F.T.); mariaida.amabile@uniroma1.it (M.I.A.); laura.giacomelli@uniroma1.it (L.G.); alessio.metere@uniroma1.it (A.M.); daniele.pironi@uniroma1.it (D.P.); giovanni.carbotta@uniroma1.it (G.C.); massimo.vergine@uniroma1.it (M.V.); massimo.monti@uniroma1.it (M.M.); 2 Department of Clinical and Experimental Medicine, University of Pisa, 56126 Pisa, Italy; alessandro.antonelli@med.unipi.it (A.A.); poupak.fallahi@med.unipi.it (P.F.); 3 Department of Internal Medicine and Medical Specialties, Sapienza University of Rome, 00161 Rome, Italy; eleonora.darmiento@fastwebnet.it; 4 Department of Surgery, S. Kliment Ohridski University, 1504 Sofia, Bulgaria; a.cornacchini@tiscali.it

**Keywords:** thyroid cancer, programmed cell death 1, programmed cell death ligand 1, diagnosis, prognosis, therapy

## Abstract

The new immunotherapy targeting the programmed cell death 1 (PD-1) receptor and its cognate ligand PD-L1 has renewed hopes of eradicating the most difficult human cancers to treat. Among these, there are the poorly differentiated and anaplastic thyroid cancers, unresponsive to all the therapies currently in use. In the present review we will summarize information regarding the expression of PD-L1 in the different thyroid cancer histotypes, its correlation with clinicopathological features, and its potential prognostic value. Then, we will evaluate the available data indicating the PD-1/PD-L1 axis as a promising target for thyroid cancer therapy.

## 1. Thyroid Cancer: An Overview

Thyroid cancer represents the most common endocrine malignancy and the fifth most common cancer in women in the United States [1]. Its annual incidence has tripled over the last twenty years, with an average annual rate of 21.4% in female, and of 7.3% in male in the years 2011–2015 [1,2,3]. Most thyroid neoplasms are well-differentiated thyroid cancers (WDTC) derived from epithelial follicular cells, comprising the papillary thyroid carcinoma (PTC) and the follicular thyroid carcinoma (FTC) histotypes, which may progress towards the poorly differentiated thyroid carcinoma (PDTC) and the anaplastic thyroid carcinoma (ATC) [4]. Although originating from the same cell type, thyroid cancers display different morphological features, functional behavior, and grade of differentiation as a result of heterogeneous genetic alterations [4,5,6,7]. Among these, the most frequent are activating point mutations of the *BRAF* and *RAS* oncogenes, and chromosomal translocations of the *RET* (REarranged during Transformation) and *NTRK1* (Neurotrophic Tyrosine Kinase Receptor 1) genes, which lead to the activation of a common carcinogenic pathway, i.e., the MAPK/ERK signaling [8,9].

Thyroid nodules are very common, affecting 19% to 67% of the adult population, but only about 5% of them harbor a malignant lesion [10]. To date, fine-needle aspiration cytology (FNAC) represents the main diagnostic tool for the evaluation of both palpable and non-palpable thyroid nodules [10,11,12]. However, in the case of indeterminate atypia or follicular proliferation, FNAC fails to discriminate adenomas from FTC or follicular variants of PTC (FVPTC), which implies the overtreatment of many patients subjected to unnecessary thyroid resection. Different molecular diagnostic approaches have been attempted to overcome this inherent limitation of FNAC and to refine preoperative diagnosis [11,12]. In recent years, the improvement of knowledge concerning the molecular changes underlying thyrocyte malignant transformation, along with remarkable progresses of high-throughput genotyping techniques, has allowed the introduction of different molecular diagnostic tests with increasingly satisfying performances, e.g., ThyroSeq, Afirma, Rosetta GX Reveal [12]. Molecular markers have also been evaluated for prognostic purposes, in order to ameliorate the stratification of patients in follow-up. Actually, the prognosis of thyroid cancer is largely favorable, with 5-year-survival rates of close to 100% for low-stage (I and II) WDTC, 90% for stage III PTC, 70% for stage III FTC, and 50% for stage IV [1,2,3,10]. However, the current TNM staging systems make a coarse prediction of recurrence or mortality risk, including patients in the same stage with considerably different disease-free survival and overall survival [13,14,15]. To this end, the European (ETA) and the American Thyroid Associations (ATA) proposed new guidelines to estimate the risk of recurrences in which TNM parameters are combined with additional clinical features such as histological variants, multifocality, outcome of post-ablative whole-body scan, vascular invasion, extrathyroidal extension, and serum thyroglobulin levels [15,16]. Despite this, patients within the same risk group still show diverse behavior in terms of disease-free interval. Recently introduced mutational markers offer high sensitivity and specificity in identifying high-risk thyroid cancers. Interestingly, the same multigene panels used to detect tumor-associated genetic alterations in thyroid FNA, like ThyroSeq, are also able to categorize a small subset of thyroid cancers with the most unfavorable outcomes, providing cancer risk stratification even before surgery [12]. This novel approach is promising, although it needs extensive validation on large case studies and integration with clinical parameters of prognostic relevance.

Lastly, an important issue still to be solved is the treatment of patients affected by advanced or undifferentiated thyroid cancers, which are more prone to disease recurrences and cancer-related deaths because refractory to adjuvant therapy with ^131^I [1,2,3,10,17,18,19]. In these patients, external beam radiation and chemotherapy do not elicit effective therapeutic responses, and thus new therapeutic strategies aimed at eradicating aggressive thyroid tumors are urgently needed [7,17,20,21,22,23].

## 2. Dysregulation of the Immune System in Thyroid Cancer

According to the Cancer Immunoediting Hypothesis, tumor infiltration by cells of the innate and adaptive immune systems reflects a physiological process aimed at eliminating malignant cells, in early as well as in advanced tumors and in metastases [24]. The ability of cancer cells to avoid the immune damage by disabling components of the host immune system is now considered a hallmark of cancer [25]. In particular, it has been shown that the immune response is turned off by a variety of mechanisms, including: i) inactivation of cytotoxic T lymphocytes (CTL) and natural killer (NK) cells by secreting immunosuppressive factors (i.e., TGF-β, indoleamine 2,3-dioxygenase, IL-10 and VEGF); ii) recruitment of immunosuppressive cells such as myeloid-derived suppressor cells and regulatory T cells (Treg); iii) expression of inhibitory ligands for the immune checkpoint receptors CTLA-4 (cytotoxic T lymphocyte antigen 4) and PD-1 (programmed cell death 1), present on the surface of activated T lymphocytes [26,27,28,29,30,31,32].

WDTCs are supposed to be poorly immunogenic because of their low mutational burden due to low neoantigen expression [33]. However, they are infiltrated by several host immune cells, including NK, tumor-associated macrophages, mast cells, dendritic cells, B and T lymphocytes [34,35,36,37,38,39,40,41,42,43,44,45,46,47,48,49,50,51]. Malignant thyrocytes are able to counteract these immune cells in different ways, for example, by inducing T-lymphocyte anergy, recruiting Treg, stimulating formation of tolerogenic antigen presenting cells (APC), downregulating neoantigen recognition, and expressing immune checkpoint molecules. The intratumoral density of immune cells, along with the expression of immunosuppressive markers, have been correlated with the thyroid differentiation score (TDS) of tumors and the BRAF status. Namely, low TDS and BRAF^V600E^ mutation were found to entail enrichment of dendritic cells, Treg, macrophages, and mast cells in PTC, together with higher expression levels of CTLA-4 and PD-L1 [36,52]. The immuno-suppressive environment of thyroid cancers is sustained also by indoleamine 2,3-dioxygenase production by tumor cells, associated with an increased Treg infiltrate and more aggressive clinicopathological features, such as extra-thyroidal extension or multifocality [33]. In essence, the ensemble of molecular mechanisms that modulate host immune cells within thyroid tumor microenvironment has immune escape as the final result, whose degree was shown to correlate with more aggressive tumor behavior [34,35,36,37,38,39,40,41,42,43,44,45,46,47,48,49,50,51].

In the present review, we’ll focus on the available data regarding the expression of PD-L1 and PD-L2 in thyroid cancer tissues and its possible clinical implications in terms of prognosis and therapy for the most aggressive thyroid cancers.

## 3. Programmed Cell Death 1 (PD-1) and Its Ligands

**Figure 1:**
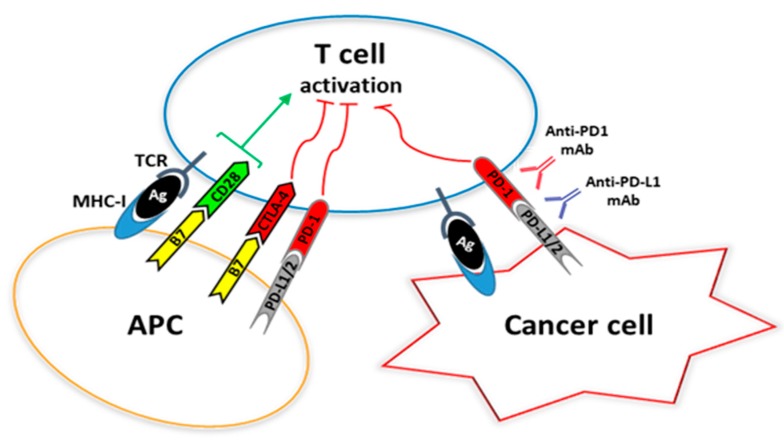
Inhibitory effect of the immune checkpoint receptors CTLA4 and PD-1 on T cell activation. T-cell activation depends on two simultaneous signals: a) interaction of the T-cell receptor with the complex MHC-antigenic peptides; b) co-stimulation by CD28 binding to CD80/CD86. The alternative ligation of CD80/CD86 to CTLA4, as well as the interaction between PD-1 and PD-L1/L2, lead to T-cell exhaustion. TCR, T cell receptor; MHC-I, major histocompatibility complex class I; CTLA-4, Cytotoxic T-Lymphocyte Antigen 4; PD-1, programmed cell death 1; PD-L1/2, PD-1 ligand 1 and 2; APC, Antigen presenting cells.

The PD-1, also known as cluster of differentiation 279 (CD279), is a type I transmembrane protein member of 288 amino acids encoded by the *PDCD1* gene localized on chromosome 2q37.3 (NCBI Gene ID: 5133) [53,54]. The PD-1 is a member of the B7-CD28 immunoglobulin superfamily, and it is expressed by T and B lymphocytes and by NK cells following activation [55]. Specifically, T cell activation is based on the interaction of the T cell receptor (TCR) with MHC molecules presenting the antigen, and is tightly regulated by different costimulatory molecules which can either potentiate or inhibit T cell response, including the CD28, the cytotoxic T-lymphocyte-associated protein 4 (CTLA-4) and the PD-1 (Figure 1) [55].

Both CTLA-4 and PD-1 exert an inhibitory action on T cells, which is thought to prevent autoimmunity and to reduce collateral tissue damage by restraining the immune reaction in chronic infections, but it is also implicated in tumor-induced immunosuppression [55]. CTLA-4 shares the same ligands of CD28, namely the B7-1 (CD80) and the B7-2 (CD86). However, upon binding to these molecules, CD28 triggers a strong costimulatory signal for T cell activation, while CTLA-4 behaves as a potent inhibitor. Two different type I transmembrane proteins, structurally related to the B7 family, bind to the PD-1 receptor, namely the PD-L1 (B7-H1 or CD274) and the PD-L2 (B7-DC or CD273) [54,55]. The *PD-L1 (CD274)* gene is located at the chromosome 9p24.2 and encodes a 290 aa protein of 33.3 kDa (NCBI Gene ID: 29126), while the *PD-L2 (PDCD1LG2)* gene is located at the chromosome 9p24.1 and encodes a 273 aa protein of about 31 kDa (NCBI Gene ID: 80380). PD-L1 and PD-L2 have been described in various healthy cells and organs, e.g., lung, heart, bladder, vascular endothelium, spleen, mesenchymal stem cells, pancreatic islets, astrocytes, neurons, and keratinocytes, although with distinct expression patterns. PD-L1 has also been detected in immune-privileged sites like the eye and the placenta, where it increases from the fourth month of gestation [56]. Among hematopoietic cells, PD-L1 is constitutively expressed on T and B lymphocytes, dendritic cells, macrophages, mesenchymal stem cells and bone marrow-derived mast cells [57]. In contrast, PD-L2 expression is restricted to activated dendritic cells, macrophages, bone marrow-derived mast cells, and the majority of peritoneal B1 cells [57]. During infection or inflammation, PD-1 and its ligands are engaged in regulating the extent of immune responses through inhibition of TCR-mediated lymphocyte proliferation, blockade of cytokine secretion, and induction of naive T cell to differentiate in Treg. Furthermore, the PD-1/PD-L pathway plays a key role in the maintenance of peripheral tolerance, hampering detrimental actions of self-reactive T cells escaped negative selection in the thymus [56]. Actually, abnormal activation of the PD-1/PD-L interaction appear to be of major clinical relevance in several autoimmune diseases, such as diabetes mellitus type I, systemic lupus erythematosus, autoimmune encephalomyelitis, inflammatory bowel disease, rheumatoid arthritis, myasthenia gravis, and autoimmune hepatitis [56]. PD-1/PD-L pathway is also considered a fundamental player of host immune escape that induces suppression of TCR-mediated activation and inhibits T cell cytolysis. Additionally, emerging evidence suggests an anti-apoptotic role of cytoplasmic PD-L1, which may confer a growth advantage to cancer cells independently from its immune suppression role. This property of PD-L1 was discovered starting from the observation that resveratrol, a polyphenol compound endowed with anticancer effects, can induce p53-dependent apoptosis in malignant cells by a mechanism that is jammed by PD-L1 upregulation. In particular, resveratrol causes nuclear accumulation of cyclooxygenase-2 (COX-2), as well as activation and nuclear translocation of mitogen-activated protein kinases (ERK1/2). COX-2 complexes with ERK1/2 and p53, and binds to promoters of certain p53-responsive genes initiating apoptosis [58]. Cytoplasmic accumulation of PD-L1 leads to retention of COX-2 in the cytoplasm, thus preventing its pro-apoptotic action [59].

## 4. Expression and Clinical Utility of PD-1 Ligands in Thyroid Cancer

Over the last few years, the expression of PD-L1 and PD-L2, mainly PD-L1, in different thyroid cancer histotypes has been investigated by several studies [37,41,42,43,44,45,46,60,61,62,63,64,65,66,67,68]. As reported in Table 1, PD-L1 protein has been assessed by means of immunohistochemistry (IHC) [37,42,43,44,45,46,62,65,66,67,68], while few studies evaluated PD-L1 at the mRNA level (Table 2) [41,42,63,64]. The results obtained indicate that routine measurement of PD-L1 expression in thyroid cancer specimens could turn useful for both patient’s diagnosis and prognosis, as well as for the identification of patients that could benefit from anti-PD-1/PD-L1 therapies.

### 4.1. PD-L1 Expression and Thyroid Cancer Diagnosis

The majority of studies examining PD-1 ligands in thyroid cancer have been aimed at evaluation of their prognostic relevance by correlating the expression levels with patients’ clinicopathological features, while some studies have attempted to estimate the diagnostic value of PD-1 ligands in thyroid cancer [42,69,70,71]. Cunha and colleagues, by means of IHC analysis of 293 DTC, 114 benign thyroid lesions and 5 normal tissues, found that PD-L1 protein did not have a diagnostic utility [42]. However, more recently, it has been reported that PD-L1 expression may help to distinguish aggressive forms of encapsulated FVPTC (EFVPTC) from the noninvasive ones, reclassified as non-invasive follicular thyroid neoplasm with papillary-like nuclear features (NIFTP) at low risk of malignancy [69,70,71,72]. In particular, Fu and colleagues retrospectively analyzed the expression of PD-L1, by means of IHC, in 52 NIFTP and 45 invasive EFVPTC in comparison with 40 benign nodules [66,67]. The authors found no significant differences in PD-L1 cytoplasmic staining between NIFTP and thyroid benign lesions, while a considerable increase was observed between NIFTP or benign lesion and invasive FVPTC [69,70,71]. Since the majority of NITPF are diagnosed as indeterminate lesions in clinical practice [73,74], the opportunity to discriminate NIFTP from invasive EFVPTC based on PD-L1 assessment should improve the diagnostic accuracy and the clinical management of these patients, reducing the number of those undergoing surgical overtreatment. 

### 4.2. PD-1 Ligand Expression and Thyroid Cancer Prognosis

As mentioned above, the majority of studies evaluating the expression of PD-L1 in thyroid cancer have been performed by IHC, using different tissue preparations, processing procedures, detection antibodies (clone E1L3N, ab82059, Ab174838, MABC290, 22C3 and SP142), cut-off values, and control tissues when available (i.e., benign lesions or normal matched tissues) [37,42,43,44,45,46,62,65,66,67,68]. Also, interpretations of IHC results (i.e., membranous staining and/or cytoplasmic staining of tumor cells) diverge in the different reports. In this context, it may be worth considering that in clinical trials with anti-PD-1/PD-L1 directed therapies, membranous but not cytoplasmic staining was considered in patient selection [44,75,76,77]. In fact, only the PD-L1 present on the membrane of tumor cells is theoretically active in inhibiting PD-1 positive immune cells. As a consequence, cytoplasmic staining should be considered as a negative result in PD-L1 immunodetection. On the whole, from the various studies recapitulated in Table 1 and Table 2, it emerges the absence of correlation between PD-L1 levels and clinicopathological parameters, while data regarding PD-L1 and disease-free survival (DFS)/disease-free interval (DFI) are discordant.

By analyzing clinical and biochemical data of a study comprising 507 PTC patients, available on the cBioPortal, we found a statistically significant correlation between higher levels of PD-L1 mRNA and lymph node metastasis, extrathyroidal invasion and DFS (Table 2) [8,63]. Analogously, a significant association between PD-L1 protein and DFS was observed in two large case studies (Table 1) [43,45]. It is also worth mentioning that in 4 out of 8 reports, a positive association between PD-L1 expression and the presence of BRAF^V600E^ mutation, known to induce a more aggressive tumor behavior, was noticed [8,9,37,64,67]. In agreement, Brauner and colleagues showed that thyroid cancer cell lines with BRAF^V600E^ mutation have higher baseline levels of PD-L1 mRNA compared to those harboring the BRAF wild type [78]. These findings appear to corroborate the reported ability of BRAF^V600E^ signaling to modulate the immune response [37]. In fact, besides inducing PD-L1 expression, BRAF^V600E^ has been shown in thyroid cancers to increase suppressive immune cell infiltration (Treg) [37]. A recent meta-analysis by Aghajani and colleagues described a moderate quality evidence from 4 studies, including 721 patients, which testified a significant association between PD-L1 positivity and poor survival in thyroid cancer patients, with a hazard ratio (HR) of 3.73 (C.I. 2.75–5.06) [79]. From the same meta-analysis, a significant association also emerged between increased PD-L1 and tumor recurrence [76]. These variations of PD-L1 levels do not seem to be due to increases in gene copy number, as indicated by a genetic screening of ATC and advanced DTC that documented co-amplification of *PD-L1* and *PD-L2* genes in 5 out of 196 ATC, and in none of 583 DTC analyzed [80]. Altogether, these findings point toward the PD-L1 as a possible prognostic marker useful to identify thyroid cancer patients with aggressive disease.

Regarding PD-L2 expression in thyroid tumors, to the best of our knowledge, only two studies have been performed so far [64]. One reported that PD-L2 mRNA levels increased in 35.1% and decreased 25.5% of PTC, but decreased in the majority of ATC, compared to control tissues [64]. In addition, higher expression of PD-L2 was found to be associated with the presence of the BRAF^V600E^ and lymph node metastasis, but not with other clinicopathological features or DFI [64]. The same study evidenced a positive correlation between PD-L1 and PD-L2 expression. In the second one, Bastman and colleagues analyzed the expression of PD-L2 mRNA in a case study consisting of 92 DTC, and found no association between PD-L2 mRNA level and tumor size or lymph node metastasis [41]. In any case, further investigations on larger case studies and examination of protein levels are required to clarify the role of PD-L2 in thyroid cancer progression and its clinical utility.

### 4.3. Anti-PD-1/PD-L1 Directed Therapies and Thyroid Cancer

Over the last decade, the considerable increase of our knowledge about mechanisms underlying the ability of cancer cells to elude the detrimental action of the immune system has led to a renewed interest for immune-based therapy in oncology, especially for hard-to-treat cancers, including the poorly differentiated and frankly anaplastic thyroid cancers [74,81]. In particular, the recognition that cancer cells express on their plasma membrane immune checkpoint molecules, such as PD-1 and CTLA-4 ligands, has headed to the generation of monoclonal antibodies capable of preventing tumor-induced exhaustion of infiltrating lymphocytes. Several antibodies targeting the PD-1/PD-L1 pathway, reported in Table 3, have been approved by the Food and Drug Administration for the treatment of multiple cancer types, including melanoma, non-small cell lung cancer, Hodgkin’s lymphoma, renal cell carcinoma, gastric and urothelial bladder cancers, and Merkell cell carcinoma [82,83].

The objective response rate to PD-1/PD-L1 directed therapies varies from 13 to 43% in different tumor types [83]. The best responses are generally observed in patients with high tumor expression of PD-L1 and high numbers of tumor-infiltrating immune cells, particularly CD8+ T cells [83,84,85,86]. In this context, the recent observations by Kim and colleagues are of particular interest, whereby, by means of an immune gene signature comprising the *PD-L1* and *CTLA-4* genes, the canonical BRAF-like and RAS-like PTC were classified into 2 subgroups each: BRAF-IR (immunoreactive) and RAS-IR, characterized by up-regulation of immune-related genes and tumor infiltration by several immune cell subtypes, including T cells; and BRAF-ID (immunodeficient) and RAS-ID, characterized by low expression of immune-related genes and low infiltration of immune cells [63,87]. Confirming the studies mentioned above, the authors showed that BRAF-IR PTC had higher expression of CTLA-4 and PD-L1, which renders this PTC subgroup a potential candidate for immune checkpoint therapies [87].

At present, no information from clinical trials with anti-PD-1/PD-L1 directed therapies enrolling patients affected by aggressive thyroid cancers is available. However, different preclinical studies demonstrated the efficacy of PD-1/PD-L1 axis blockade in restraining growth of ATC-derived cell lines injected in mice [78,88,89]. In particular, it has been shown that simultaneous administration of an anti PD-L1 antibody (10F.9G2) and a BRAF inhibitor (PLX4720) or lenvatinib (multitargeted tyrosine kinase inhibitor of VEGFR1-VEGFR3, FGFR1-FGFR4, PDGFRα and RET) synergistically reduced tumor volume in an immunocompetent murine model bearing implanted syngeneic ATC [78,88]. In these experiments, it was also found that treatment with anti-PD-L1 antibody resulted in an increased tumor infiltration of CD8+ T cell with augmented cytotoxic profile [78,88]. Kollipara and colleagues reported the encouraging case of a 62-year-old male who, following ATC diagnosis, was initially treated by thyroidectomy with lymph node dissection [90]. Subsequently, the positron emission tomography (PET) revealed the presence of a mass in the thyroid bed, and metastases in the supraclavicular region as well as in the upper lobes of right and left lungs. The patient was then treated with doxorubicin and cisplatin, to which he was unresponsive, as judged by the progression of lung metastases, and a second-line paclitaxel treatment was equally ineffective. Following the identification of the BRAF^V600E^ mutation and PD-L1 protein by IHC in tumor tissue, the patient was treated with vemurafenib (BRAF inhibitor) and nivolumab. Whereupon, the patient experienced a continued reduction of the metastatic lesions with complete radiographic and clinical remission of the disease 20 months after the beginning of nivolumab therapy [90]. In another study, performed at the MD Anderson Cancer Center, 12 ATC patients in treatment with different kinase inhibitors (5 of which were treated with lenvatinib, 6 with dabrafenib plus trametinib, and 1 with trametinib alone) started to receive pembrolizumab in combination with kinase inhibitors at the time of disease progression [91]. Of these patients, 5 (42%) had a partial response, 4 (33%) exhibited stable disease, and 3 (25%) had progressive disease [91]. Very recently, the results of a phase 1B clinical trial (NCT02054806) evaluating the efficacy of pembrolizumab monotherapy in patients with PD-L1-positive advanced DTC have been reported [92]. Twenty-two patients were enrolled in the study and treated with pembrolizumab at the dose of 10 mg/Kg administered every two weeks up to 24 months. Eighteen patients (82%) had low-grade treatment-related adverse effects, including diarrhea (32%), fatigue (18%) and rash (14%), but no patient was discontinued due to deleterious side effects [92]. A partial response to treatment was observed in 2 patients (overall response rate of 9%), 13 patients experienced a stable disease (59%), while 7 had a progressive disease [92]. At the moment, there is much interest in the results of an ongoing phase II clinical trial evaluating pembrolizumab on metastatic or locally advanced ATC patients, that should be completed by October 2019 (NCT02688608) [93]. This is a multi-center, open-label trial estimated to enroll at least 20 ATC patients, aimed at assessing the therapeutic effects of pembrolizumab administered at 200 mg intravenously every 3 weeks for up to 18 months.

Since the activation of the MAPK signaling in thyroid cancer has been shown to negatively modulate the immune response in the tumor microenvironment, it is likely that targeted therapies directed against this pathway may revert the immune suppression due to abnormal kinase activation [94]. For example, Sorafenib, a multitargeted antiangiogenic tyrosine kinase inhibitor, was shown to reduce Treg numbers and to inhibit their function in an orthotopic mouse model of hepatocellular carcinoma [95]. Thus, a strategy that could be worth exploring in approaching thyroid cancer is the combination of immune and targeted therapies [90,94]. In this regard, a phase 1b/2 clinical trial (NCT02501096) employing pembrolizumab plus lenvatinib is currently underway with patients affected by selected solid tumors, including thyroid cancers. The study is estimated to be completed by February 2020 [88,94,95,96,97].

## 5. Conclusions

The information so far available suggests that PD-L1 could represent a useful prognostic marker for risk stratification of thyroid cancer patients, and that anti-PD-1/PD-L1 directed therapies could be a valid option for patients affected by the most aggressive thyroid cancers, such as PDTC and ATC, unresponsive to the currently available therapies. Some issues, however, still remain to be addressed. In particular, the standardization of the IHC techniques, the interpretation of PD-L1 immunoreactivity in cancer tissues, and a more reliable characterization of biomarkers capable of predicting patients’ response to anti PD-1/PD-L1 therapies.

## Figures and Tables

**Table 1:** Association of PD-L1 protein levels with clinico-pathological features and BRAF mutational status in thyroid cancer patients. ETI, extra-thyroidal invasion; DTC, differentiated thyroid cancer; PTC, papillary thyroid cancer; ATC, anaplastic thyroid cancer; FTC, follicular thyroid cancer; PDTC, poorly differentiated thyroid cancer; --, non-evaluated; DFS, disease-free survival; MAB, monoclonal antibody; PAB polyclonal antibody.

					Correlations/Associations									
			Antibodies		Age	Gender	T	N	M	Stage	Multifocality	ETI	DFS	BRAF^V600E^
Ref. No.	Case Study	PD-L1 Expression	Host-Type	Clone (Source)										
[42]	407 patients, Including 293 DTC	Increased levels in DTC vs. benign lesions	Rabbit PAB	Ab82059 (Abcam)	No	No	No	No	No	No	No	No	--	--
[37]	33 PTC	Increased levels in BRAF^V600E^ vs. BRAF^wt^ PTC	Rabbit PAB	4059 (ProSci)	--	--	--	--	--	--	--	--	--	Yes
[61]	13 ATC	Positive in 23% of ATC patients	Mouse MAB	5H1 (non-commercial)	--	--	--	--	--	--	--	--	--	--
[43]	251 patients including 185 PTC	Increased in PTC vs. benign lesions	Rabbit MAB	E1L3N (Cell Signaling)	--	--	--	--	--	No	No	No	Yes	--
[41]	92 DTC; 22 patients with advanced DTC/ATC	Positive in 64% of DTC and 59.1% of advanced DTC/ATC	Rabbit MAB	SP142 (Spring Bioscience)	--	--	No	Yes	--	--	--	--	--	No
[44]	407 thyroid cancers	Positive in 6.1% of PTC, 7.6% of FTC, 22.2% of ATC	Rabbit MAB	SP142 (Spring Bioscience)	No	No	No	No	No	No	No	No	No	No
[45]	260 PTC and normal matched tissues	Increased in 52.3% of PTC vs. normal tissue	Rabbit MAB	Ab174838 (Abcam)	No	No	No	No	--	--	Yes	Yes	Yes	--
[62]	126 PTC	Positive in 53.2% of PTC	Rabbit MAB	SP142 (Spring Bioscience)	No	Yes	No	No	--	No	No	--	--	No
[46]	49 ATC	Positive in 28.6% of ATC	Rabbit MAB	E1L3N (Cell Signaling)	--	--	--	--	--	--	--	--	--	No
[68]	16 ATC	Positive in 81.3% of ATC	Rabbit MAB	E1L3N (Cell Signaling)	No	No	--	--	--	No	--	--	No	--
[66]	75 PTC	Positive in 66.7% of PTC	Mouse MAB	22C3 (DAKO)	No	No	No	No	--	No	No	Yes	No	--
[65]	28 PDTC	Positive in 25% of PDTC	Rabbit MAB	E1L3N (Cell Signaling)	No	No	Yes	--	No	No	Yes	No	No	--
[67]	110 PTC	Positive in 46% of PTC	Rabbit MAB	SP142 (Spring Bioscience)	No	No	No	No	--	No	No	--	--	Yes

**Table 2:** Association of PD-L1 mRNA levels with clinico-pathological features and BRAF mutational status in thyroid cancer patients. ETI, extra-thyroidal invasion; DTC, differentiated thyroid cancer; PTC, papillary thyroid cancer; ATC, anaplastic thyroid cancer; --, non-evaluated; DFS, disease-free survival.

			Correlations/Associations									
			Age	Gender	T	N	M	Stage	Multifocality	ETI	DFS	BRAF^V600E^
Ref. No.	Case Study	PD-L1 mRNA Expression										
[42]	407 patients, Including 293 DTC	Increased levels in DTC vs. benign lesions	Yes	No	No	No	No	Yes	No	No	--	--
[63]	482 PTC and 58 normal tissues	Unvaried in PTC vs. normal tissues	No	Yes	No	Yes	No	No	--	Yes	Yes	Yes
[41]	92 DTC; 22 advanced DTC/ATC	Positivity in 64% of DTC and 59.1% of advanced DTC/ATC	--	--	No	Yes	--	--	--	--	--	No
[64]	94 PTC and normal matched tissues, 11 ATC	Increased in 46.8% of PTC and 27.3% of ATC	No	No	No	No	--	No	--	--	Yes	Yes

**Table 3:** FDA approved PD-1/PD-L1 inhibitors. HNSCC, head and neck squamous cell carcinoma; NSCLC, non-small cell lung cancer; cHL, classical Hodgkin lymphoma; MSI-H, microsatellite instability-high; PMBCL, primary mediastinal large B-cell lymphoma; HCC, hepatocellular carcinoma; SCCHN, squamous cell carcinoma of the head and neck; RCC, renal cell carcinoma; SCLC, small cell lung cancer; CSCC, cutaneous squamous cell carcinoma. Source: www.fda.gov.

Name	Commercial Name (Company)	IgG Isotype	Target	FDA Approval	
				Year	Cancer Type
Pembrolizumab	Keytruda (Merck)	IgG4	PD-1	2014	Melanoma
				2016	HNSCC, NSCLC
				2017	Gastric/gastroesophageal adenocarcinoma, cHL, urothelial carcinoma, MSI-H cancers
				2018	Merkel cell carcinoma, PMBCL, HCC, cervical cancer
Nivolumab	Opdivo (Bristol-Myers Squibb)	IgG4	PD-1	2014	Melanoma, NSCLC
				2016	SCCHN, cHL
				2017	Urothelial carcinoma, HCC, MSI-H colorectal cancer
				2018	RCC, SCLC
Cemiplimab-rwlc	Libtayo (Regeneron Pharmaceuticals)	IgG4	PD-1	2018	CSCC
Atezolizumab	Tecentriq (Genentech Oncology)	IgG1	PD-L1	2016	NSCLC, urothelial carcinoma
Avelumab	Bavencio (EMD Serono)	IgG1	PD-L1	2017	Merkel cell carcinoma, urothelial carcinoma
Durvalumab	Imfinzi (AstraZeneca)	IgG1k	PD-L1	2017	Urothelial carcinoma
				2018	NSCLC

## Abbreviations

WDTC	Well-Differentiated Thyroid Cancers
PTC	Papillary Thyroid Cancer
FTC	Follicular Thyroid Cancer
PDTC	Poorly Differentiated Thyroid Cancers
ATC	Anaplastic Thyroid Cancer
FNAC	Fine-Needle Aspiration Cytology
TNM	Tumor Node Metastasis
CTL	Cytotoxic T Lymphocytes
NK	Natural Killer
PD-1	Programmed Cell Death 1
TGF-TGF-β	Transforming Growth Factor β
IL-10	Interleukin 10
VEGF	Vascular Endothelial Growth Factor
CD	Cluster of Differentiation
CTLA-4	Cytotoxic T-Lymphocyte Antigen 4
DFS	Disease-free survival
DFI	Disease-free interval
ORR	Overall response rate
